# Major multinational food and beverage companies and informal sector contributions to global food consumption: implications for nutrition policy

**DOI:** 10.1186/1744-8603-7-26

**Published:** 2011-08-01

**Authors:** Eleanore Alexander, Derek Yach, George A Mensah

**Affiliations:** 1 Global Health and Agriculture Policy, PepsiCo, Inc., Purchase, NY, USA; 2 Global Nutrition, Global Research and Development, PepsiCo, Inc., Purchase, NY, USA

## Abstract

**Background:**

In recent years, 10 major multinational food and beverage companies have worked together within the International Food and Beverage Alliance (IFBA) to increase their commitments to public health. Current IFBA commitments include initiatives to improve the nutrition quality of products and how these products are advertised to children. The impact and magnitude of IFBA member contributions to the total market share of packaged foods and beverages consumed remain incompletely understood, however.

**Methods:**

In order to evaluate this impact, we examined packaged food and soft drink company shares provided by Euromonitor, an international independent market analysis company. Packaged foods include baby food, bakery, canned/preserved food, chilled/processed food, confectionery, dairy, dried processed food, frozen processed food, ice cream, meal replacement, noodles, oils and fats, pasta, ready meals, sauces, dressings and condiments, snack bars, soup, spreads, and sweet and savoury snacks. Soft drinks include carbonates, packaged fruit/vegetable juice, bottled water, functional drinks, concentrates, ready-to-drink tea, ready-to-drink coffee and Asian specialty drinks. We calculated the market shares for IFBA companies, globally and within nine countries--the US, China, India, Egypt, South Africa, Brazil, Mexico, Turkey and the UK.

**Results:**

Worldwide, the top ten packaged food companies account for 15.2% of sales, with each individual company contributing less than 3.3%. The top ten soft drink companies account for 52.3% of sales worldwide; Coca-Cola and PepsiCo lead with 25.9% and 11.5% of sales, respectively.

**Conclusions:**

Although the top ten soft drink companies account for half of global sales, the top ten packaged food companies account for only a small proportion of market share with most individual companies contributing less than 3.3% each. Major multinational companies need to be joined by the myriad of small- and medium-sized enterprises in developing and implementing programs to improve the health of the public, globally. Without full participation of these companies, the impact of commitments made by IFBA members and other major multinational food and beverage companies will remain limited.

## Introduction

The packaged food and beverage industry, including large multinationals (MNCs)^1^, medium- and small-sized enterprises (SMEs)^2 ^as well as the informal sector, need to be involved in improving the nutrition status of the populations they serve [1]. Major food and beverage companies--Ferrero, General Mills, Grupo Bimbo, Kellogg's, Kraft Foods, Mars, Nestlé, PepsiCo, the Coca-Cola Company and Unilever--have worked together over several years within the International Food and Beverage Alliance (IFBA) to increase their commitment to public health [2]. The IFBA set five global commitments addressing food reformulation, consumer information, responsible marketing, promotion of healthy lifestyles and public private partnerships. Progress includes pledges to improve the nutrition quality of products and restrict advertising to children. However, the impact and magnitude of the IFBA contribution to the total market share of packaged foods and beverages consumed is not fully understood due to insufficient data.

The data on the relative contribution of various food and beverage companies to people's diets is critical, as they often drive policy development to improve nutrition status. Basic information, for example on sources of sodium, is needed for rationale in policy development. Without such information, anecdote can drive policies in ways that may undermine public health goals. This paper seeks to fill one gap in knowledge by describing which companies are the major contributors to packaged food and soft drink sales, both at a global level and within nine countries--the US, China, India, Egypt, South Africa, Brazil, Mexico, Turkey and the UK.

### Objectives

This paper defines the contribution of selected players in the food and beverage industry to the sales of packaged foods and soft drinks sold globally and in selected countries. Further, it describes food and soft drink company pledges related to health, with a focus on IFBA commitments.

## Methods

Data were gathered through Euromonitor International, an independent market analysis company that provides information on industries, countries and consumers, using Passport, a global market analysis software platform [3]. Euromonitor gathers data using national- and international-level desk research, including company research and analysis, store checking, trade interviewing with national players, market analysis and MNC research and analysis. Data standardization ensures international comparability across the global database.

Data were extracted for the world, and for the following countries: US, China, India, Egypt, South Africa, Brazil, Mexico, Turkey and the UK. Countries were selected based on market strength or recent market growth. Leading companies were ranked within the top ten for each geographic region based on sales value.

### Categories: Packaged Food and Soft Drinks

Packaged food^3 ^data are defined in terms of retail sales and foodservice sales [Table 1] [3]. Retail sales include sales intended for consumption at home. Foodservice is defined as sales to consumers in a non- or semi-captive environment and includes venues such as cafes, bars and street stalls. Packaged food includes sales by corporations, retailers as "private label," artisanal and generic/unbranded. Artisanal products are those sold on the site of production and are common in bakery products.

**Table 1:** Packaged food and soft drink definitions

Packaged food	baby food
	bakery
	canned/preserved food
	chilled/processed food
	confectionery
	dairy
	dried processed food
	frozen processed food
	ice cream
	meal replacement
	noodles
	oils and fats,
	pasta
	ready meals
	sauces
	dressings and condiments
	snack bars
	soup
	spreads
	sweet and savoury snacks.
Soft drinks	Carbonates
	packaged fruit/vegetable juice
	bottled water
	functional drinks
	concentrates
	ready-to-drink tea
	ready-to-drink coffee
	Asian specialty drinks

Soft drink company share data are defined in off-trade value. Off-trade does not include sales through bars, restaurants and cafes. Products in the soft drink category include: carbonates, packaged fruit/vegetable juice, bottled water, functional drinks, concentrates, ready-to-drink tea, ready-to-drink coffee and Asian specialty drinks [Table 1]. The terms beverage and soft drink are used interchangeably in this paper; beverage if often used to promote understanding that the category is not limited to carbonated soft drinks. Soft drink sales are categorized as sales from corporations, private label and other.

Euromonitor does not collect data on the informal sector (defined as sales that are not taxed). While products from the informal sector and artisanal products are often locally made, products from the informal sector differ from artisanal products because artisanal products are taxed, while informal sector products are not.

### Metrics

Packaged food sales are measured in percent retail value RSP (defined as retail selling prices or how much the product sells for in the store) for the year 2009. Soft drink value is measured in off-trade value RSP for the year 2010. On-trade soft drink company share value is not available from Euromonitor Passport; however, the omission of on-trade soft drink sales, often led by MNCs, limits this analysis. Most soft drink companies rank similarly in volume and value. Packaged food company share data are only available for value. Therefore, company shares value data are reported in this paper.

## Results

[See Tables 2 and 3 for country specific packaged food and soft drink data]

**Table 2:** Country Packaged Food Company Shares (Euromonitor, 2011)

US Packaged Food Company Shares				
**Rank**	**Company**	**Value (%)**	**% top 10**	**Artisanal (%)**
			31.9	25.9
**1**	Kraft Foods Inc	6.7		
**2**	PepsiCo Inc	5.3		
**3**	Nestlé SA	3.7		
**4**	Mars Inc	3		
**5**	General Mills Inc	2.6		
**6**	Kellogg Co	2.6		
**7**	Hershey Co, The	2.2		
**8**	ConAgra Foods Inc	2.1		
**9**	Unilever Group	2		
**10**	Campbell Soup Co	1.7		

**China Packaged Food Company Shares**				

**Rank**	**Company**	**Value (%)**	**% top 10**	**Artisanal (%)**
			25.4	7.1
**1**	Inner Mongolia Mengniu Dairy Industry (Group) Co Ltd	4.5		
**2**	Inner Mongolia Yili Industrial Group Co Ltd	4.1		
**3**	Kuok Oils & Grains Pte Ltd (KOG)	3.3		
**4**	Ting Hsin International Group	2.9		
**5**	Shineway Group	2.8		
**6**	Hangzhou Wahaha Group	2.1		
**7**	Want Want Group	1.8		
**8**	Bright Food (Group) Co Ltd	1.6		
**9**	Mars Inc	1.2		
**10**	Nestlé SA	1.1		

**India Packaged Food Company Shares**				

**Rank**	**Company**	**Value (%)**	**% top 10**	**Artisanal (%)**
			39.6	4.2
**1**	Gujarat Co-operative Milk Marketing Federation Ltd	8		
**2**	National Dairy Development Board	4.9		
**3**	Nestlé SA	4.8		
**4**	Britannia Industries Ltd	4.4		
**5**	Parle Products Pvt Ltd	4.2		
**6**	Karnataka Cooperative Milk Producers Federation Ltd	2.9		
**7**	Cadbury Plc	2.9		
**8**	GlaxoSmithKline Plc	2.8		
**9**	Ruchi Group	2.4		
**10**	Tamil Nadu Cooperative Milk Producers Federation Ltd	2.3		

**Egypt Packaged Food Company Shares**				

**Rank**	**Company**	**Value (%)**	**% top 10**	**Artisanal (%)**
			18.8	48
**1**	Savola Group	3.6		
**2**	Nestlé SA	2.5		
**3**	Al Doha Co for Processing & Distribution	2.3		
**4**	Delta Rice SAE	1.6		
**5**	Cadbury Plc	1.6		
**6**	Americana Group	1.6		
**7**	Faragello Group	1.6		
**8**	Arma Food Industries	1.5		
**9**	Juhayna Food Industries	1.3		
**10**	PepsiCo Inc	1.2		

**South Africa Packaged Food Company Shares**				

**Rank**	**Company**	**Value (%)**	**% top 10**	**Artisanal (%)**
			51.8	7.3
**1**	Tiger Brands Ltd	17.2		
**2**	Unilever Group	4.9		
**3**	Parmalat Group	4.8		
**4**	Nestlé SA	4.6		
**5**	Clover Ltd	4.6		
**6**	Dairybelle (Pty) Ltd	4		
**7**	Pioneer Food Group Ltd	3.7		
**8**	Cadbury Plc	2.8		
**9**	AVI Ltd	2.8		
**10**	PepsiCo Inc	2.4		

**Brazil Packaged Food Company Shares**				

**Rank**	**Company**	**Value (%)**	**% top 10**	**Artisanal (%)**
			30.4	21.1
**1**	Nestlé SA	7.9		
**2**	Brf Brasil Foods SA	4.7		
**3**	Unilever Group	3.3		
**4**	Danone, Groupe	3		
**5**	PepsiCo Inc	2.4		
**6**	Kraft Foods Inc	2.1		
**7**	Bunge International Ltd	2.1		
**8**	M Dias Branco Indústria e Comércio de Alimentos	1.8		
**9**	Cadbury Plc	1.7		
**10**	Itambé SA	1.4		

**Mexico Packaged Food Company Shares**				

**Rank**	**Company**	**Value (%)**	**% top 10**	**Artisanal (%)**
			32.4	31.5
**1**	Bimbo SA de CV, Grupo	8.8		
**2**	PepsiCo Inc	5.2		
**3**	Nestlé SA	4.4		
**4**	Industrial Lala SA de CV, Grupo	3.6		
**5**	Ganaderos Productores de Leche Pura SA de CV	2.1		
**6**	Cadbury Plc	2		
**7**	Sigma Alimentos SA de CV	1.8		
**8**	Kellogg Co	1.7		
**9**	Unilever Group	1.7		
**10**	Conservas La Costeña SA de CV	1.1		

**Turkey Packaged Food Company Shares**				

**Rank**	**Company**	**Value (%)**	**% top 10**	**Artisanal (%)**
			20.7	56.9
**1**	Ülker Gida Sanayi ve Ticaret AS	7.6		
**2**	Unilever Group	3.2		
**3**	Yasar Holding AS	1.6		
**4**	Eti Gida Sanayii ve Ticaret AS	1.5		
**5**	Sütas AS	1.3		
**6**	Danone, Groupe	1.2		
**7**	Cadbury Plc	1.2		
**8**	Tat Konserve Sanayii AS	1.1		
**9**	PepsiCo Inc	1.1		
**10**	Marsan Gida San ve Tic AS	0.9		

**UK Packaged Food Company Shares**				

**Rank**	**Company**	**Value (%)**	**% top 10**	**Artisanal (%)**
			26.4	2.8
**1**	Cadbury Plc	4.5		
**2**	Mars Inc	3.7		
**3**	Premier Foods Plc	3.6		
**4**	Nestlé SA	2.7		
**5**	PepsiCo Inc	2.6		
**6**	Unilever Group	2.6		
**7**	Heinz Co, HJ	2		
**8**	United Biscuits (Holdings) Plc	1.9		
**9**	Danone, Groupe	1.4		
**10**	Dairy Crest Group Plc	1.4		

**Table 3:** Global Soft Drink Company Shares (Euromonitor, 2011)

Region	Rank	Company	Value (%)	% top 10
***World ***				52.3
	**1**	Coca-Cola Co, The	25.9	
	**2**	PepsiCo Inc	11.5	
	**3**	Nestlé SA	3	
*All other companies contribute less than 2.9%				
***US***				70.8
	**1**	Coca-Cola Co, The	24.9	
	**2**	PepsiCo Inc	22.5	
	**3**	Dr Pepper Snapple Group Inc	9.2	
*All other companies contribute less than 4.6%				
***China ***				57
	**1**	Coca-Cola Co, The	15.5	
	**2**	Tingyi (Cayman Islands) Holdings Corp	13	
	**3**	PepsiCo Inc.	5.5	
*All other companies contribute less than 5.6%				
***India ***				88.4
	**1**	Coca-Cola Co, The	33.7	
	**2**	PepsiCo Inc	24.2	
	**3**	Parle Bisleri Ltd	13.8	
*All other companies contribute less than 6.5%				
***Egypt ***				93.6
	**1**	PepsiCo Inc	34.9	
	**2**	Coca-Cola Co, The	32	
	**3**	Juhayna Dairy Corp	7	
*All other companies contribute less than 6.3%				
***South Africa ***				79
	**1**	Coca-Cola Co, The	49.8	
	**2**	Tiger Brands Ltd	9.4	
	**3**	PepsiCo Inc	5.5	
*All others contribute less than 3.5%				
***Brazil ***				67.9
	**1**	Coca-Cola Co, The	39	
	**2**	PepsiCo Inc	9.3	
	**3**	Anheuser-Busch InBev NV	5.2	
*All others contribute less than 3.3%				
***Mexico ***				88
	**1**	Coca-Cola Co, The	47.7	
	**2**	PepsiCo Inc	15.1	
	**3**	Danone, Groupe	7.5	
*All others contribute less than 3.3%				
***Turkey ***				68.8
	**1**	Coca-Cola Co, The	32.3	
	**2**	PepsiCo Inc	6.9	
	**3**	Yildiz Holding AS	6.1	
*All other contribute less than 5.6%				
***UK ***				61.2
	**1**	Coca-Cola Co, The	22.5	
	**2**	PepsiCo Inc	10.1	
	**3**	GlaxoSmithKline Plc	8.8	
*All others contribute less than 6.1%				

**Figure 1:**
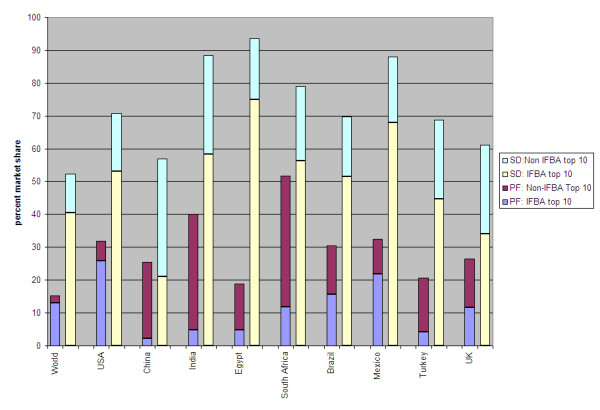
**IFBA and top ten packaged food and soft drink company shares**.

The top ten packaged food companies globally--Nestlé, Kraft Foods, Unilever, PepsiCo, Mars, Danone, Cadbury^4^, Kellogg, General Mills and Ferrero--account for 15.2% of global packaged food sales [Table 4]. Each company contributes fewer than 3.3% of total sales. IFBA members in the top ten--Nestlé, Kraft, Unilever, PepsiCo, Mars, Kellogg, General Mills and Ferrero--account for 13% of global packaged food sales [Figure 1]. Artisanal packaged food products contribute 11.3% of total sales.

**Table 4:** Global Packaged Food Company Shares 2009 (Euromonitor, 2011)

Rank	Company	Value (%)	% top 10	Artisanal (%)
			15.2	11.3
**1**	Nestlé SA	3.2		
**2**	Kraft Foods Inc	2.4		
**3**	Unilever Group	2.1		
**4**	PepsiCo Inc	1.8		
**5**	Mars Inc	1.4		
**6**	Danone, Groupe	1.3		
**7**	Cadbury Plc	0.9		
**8**	Kellogg Co	0.8		
**9**	General Mills Inc	0.7		
**10**	Ferrero Group	0.6		

**Figure 2:**
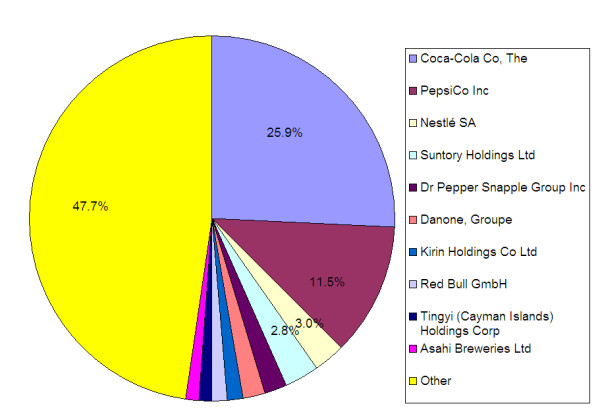
**Global Soft Drink Company Shares 2010**.

The top ten soft drink companies globally--Coca-Cola, PepsiCo, Nestlé, Suntory Holdings Ltd, Dr Pepper Snapple Group, Danone, Kirin Holdings Co Ltd, Red Bull GmbH, Tingyi (Cayman Islands) Holdings Corp and Asahi Breweries Ltd--account for 52.3% of total soft drink sales [Figure 2]. Three IFBA members--Coca-Cola, PepsiCo and Nestlé--rank in the top ten for the global soft drink market, with sales totaling 40.4% [Figure 2].

## Discussion

### IFBA companies account for a relatively small fraction of global packaged food sales, while a few IFBA companies dominate soft drink sales

Overall, individual packaged food companies each contribute a small share of total sales and IFBA member companies vary in strength by country: IFBA companies that rank in the top ten for packaged food sales range in contributions from 2.3% of sales in China to 25.9% of sales in the US. The range of soft drink company involvement in each country varies widely: IFBA members in the top ten for soft drink sales range from 21.0% in China to 75.0% in Egypt.

### Data gaps

Data are needed to describe the size of the informal sector in the packaged food and soft drink markets. Collection of data from the informal sector is difficult, as informal sector vendors mostly operate beyond the regulatory reach of governments. Further data are also needed from the informal sector and from companies on the amount of specific nutrients that each organization contributes to nutrient intake. This paper uses company shares sales data, the most specific data available for both packaged food and soft drink on Euromonitor; however, population intake data are needed to describe the nutrient contributions of companies and specific foods. The data on the nutrient contribution from each company and the informal sector will describe the role of each organization in contributing to nutrient intake.

The sales data detailed in Tables 2, 3 and 4 do not thoroughly describe the global reach of food and beverage industry MNCs. This requires detailed information about corporate ownership, partnerships, joint-ventures and local counterparts. The larger picture of corporate governance, ownership and control must be addressed to understand fully the global reach of companies.

**Figure 3:**
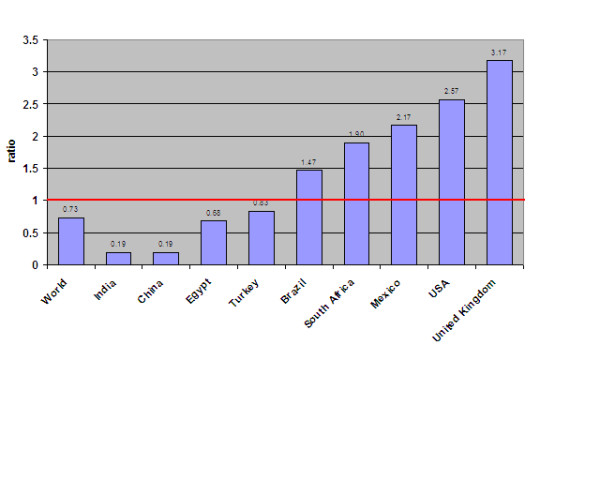
**Ratio of packaged food to fruit and vegetable market size (volume)**.

Comparing the packaged food market to the fruit and vegetable market in a country will provide insight into food and nutrient intake. Globally, the sales volume of the packaged food market is smaller than the fruit and vegetable market with a ratio of 0.73 [Figure 3]. However, five of the nine countries examined have a ratio greater than 1.0 and the magnitude of difference between the markets is striking in several countries; for example, the packaged food market is more than double the size of the fruit and vegetable market in the US and Mexico, and more than triple the size in the UK. A ratio greater than 1.0 suggests a larger contribution of packaged foods than fresh foods to the diet in these countries, and packaged foods often contain high amounts of salt and fat as well as refined flour and added sugar.

### What about the informal sector?

The packaged food and soft drink markets include a wide range of players including the formal and informal sector. Keith Hard first described the informal sector as those earning income through various activities yet not included in the census under wage employment [as cited in [4]]. Euromonitor describes the informal sector as retailing that is not taxed and overestimates the contribution of individual companies by not collecting data on the informal sector.

In some areas, especially in developing countries, the informal food sector comprises a large segment of the working population, gross national income, food processing output, as well as a significant portion of total nutrient intake [4]. Up to 60% of people in some African cities are employed through the informal food sector. The informal food sector accounts for a significant portion of food purchases and energy intake in some populations: 60% of Brazil's mini and midsize supermarkets are informal retailers and 40% of resident total energy intake in Bangkok, Thailand is accounted for by street food, including 88% of energy intake for children four to six years of age [4,5]. Street foods are common in South Africa and are purchased at almost twice the amount of fast food--fruit is the most commonly purchased item, chosen by 60% of consumers (Steyn & Labadarios: Street foods and fast foods: How much do South Africans consume?, submitted).

In addition to contributing to higher energy and nutrient intakes, foods from the informal sector may also carry a higher food safety and health risk as these foods are not subjected to the same rigorous standards required for the formal packaged food sector. Unpublished data from South Africa, for example, show higher levels of artificial food color and sodium in Bottom of the Pyramid (BoP) products than in brand-name products (PepsiCo data: RSA BoP Salty & Sweet Snacks Quality Survey). For example, BoP products contain 30% more sodium than Simba brand products, with mean sodium levels of 332 mg and 233 mg, respectively.

### Corporate Health and Wellness Initiatives: IFBA vs. Others

Members of IFBA have made substantial progress in response to the five commitments made by IFBA in 2006 [6]. Other major regional pledges include those made by the CFBAI^5 ^the EU pledge^6^, the GCC pledge^7 ^and the ICBA pledge^8 ^[7-10]. Many of these other pledges have been committed to by IFBA members, and most companies that follow the EU and IFBA pledges are large MNCs--few small and medium-sized companies are included.

Several top packaged food companies that are not IFBA members have health and wellness pledges, including Parmalat and Brasil Foods with pledges on marketing to kids, and Premier Foods with diet and health policies to improve ingredients. Conversely, most of the leading packaged food companies in China and India, and several companies in the US, South Africa, Brazil, Mexico and the UK are national companies without published evidence of significant health and wellness pledges. Additionally, numerous soft drink companies in each country are not engaged in significant health and wellness pledges.

### Context: UN High Level Meeting on Noncommunicable Diseases (NCDs)

The UN High Level Meeting on NCDs (September 19 and 20, 2011) offers the opportunity to place NCDs, specifically cardiovascular disease, diabetes, cancer and chronic lung disease, higher on government, NGO and development agency agendas. It will focus on ways to improve the four major risk factors for NCDs: unhealthy diet, tobacco use, physical inactivity and alcohol intake [11]. The draft Outcomes Document requests the private sector to reformulate foods and beverages, reduce sugar, salt, and trans-fats, and responsibly market and advertise to children. IFBA companies are actively engaged in such actions. This analysis highlights the importance of including small and medium companies, and in time, the vast informal food sector, in initiatives to improve population health. Without this active participation, IFBA gains will remain limited.

### Future Work

Further work is needed to determine whether IFBA members represent the "healthiest" part of the food system. National health and wellness pledges are needed to include companies that are not incorporated through international pledges such as the IFBA. In addition, efforts aimed at informing consumers on health issues related to nutrition through health promotion campaigns in low and middle income countries are needed and can increase demand for health and wellness policies.

The lack of data describing nutrient intake from both the formal and informal sectors of the food and beverage market highlights research gaps in defining major contributors to intake. Further data need to be collected that describe nutrient contributions of foods from the formal and informal sectors.

The lack of engagement, including research, related to the informal sector is a target area for future progress. Defining the size and impact of the informal sector is necessary to understand the best ways to engage the informal sector in actions to improve diets.

## Competing interests

EA is a PepsiCo consultant; DY and GM are fulltime PepsiCo employees.

## Authors' contributions

EA researched and contributed to writing the document; DY led conception and development of arguments and contributed to writing the document; GM critically revised the document. All authors read and approved the final manuscript.

## Appendix

^1) ^The OECD describes multinational enterprises, discussed in this paper as multinational companies or multinational corporations (MNCs), as "companies or other entities whose ownership is private, state or mixed, established in different countries and so linked that one or more of them may be able to exercise a significant influence over the activities of others and, in particular, to share knowledge and resources with the others." [1]

^2) ^Small and medium enterprises (SMEs) refer to enterprises usually with 250 or less employees who mainly operate in one country or a single well defined geographic region.

^3) ^Packaged foods, as defined by Euromonitor, include baby food, bakery, canned/preserved food, chilled/processed food, confectionery, dairy, dried processed food, frozen processed food, ice cream, meal replacement, noodles, oils and fats, pasta, ready meals, sauces, dressings and condiments, snack bars, soup, spreads, and sweet and savoury snacks.

^4) ^Cadbury became a part of Kraft in 2010; Cadbury company shares are listed separately from Kraft in 2009 packaged food data.

^5) ^The Children's Food and Beverage Advertising Initiative (CFBAI) was started in 2006 by the Council of Better Business Bureaus to allow food and beverage companies to have transparent advertising self-regulation (CFBAI, 2010).

^6) ^The European Union (EU) pledge was started in 2007 to change food and beverage advertising to children and includes eleven company members (EU Pledge, n.d.).

^7) ^Seven companies operating in the Gulf Cooperation Council for the Arab States of the Gulf (Bahrain, Kuwait, Oman, Qatar, Saudi Arabia, and the United Arab Emirates) signed a pledge in 2010 to restrict marketing and advertising to children under 12 (Yale, 2010).

^8) ^In 2009, the International Council of Beverages Associations (ICBA) implemented a pledge on marketing to children under 12; Coca-Cola and PepsiCo are members.
